# Commentary: Comparison of the effects of remimazolam and dexmedetomidine on early postoperative cognitive function in elderly patients with gastric cancer

**DOI:** 10.3389/fnagi.2024.1487104

**Published:** 2024-11-27

**Authors:** Ye-fang Yang, Xu-zhou Dang, Wen-jie Zhang

**Affiliations:** ^1^College of Anesthesia, Shanxi Medical University, Taiyuan, China; ^2^Department of Anesthesiology, First Hospital of Shanxi Medical University, Taiyuan, China

**Keywords:** cognitive function, remimazolam, dexmedetomidine, elderly, gastrectomy for gastrointestinal cancer

## 1 Introduction

An article by Yong Qing Liao and colleagues titled “*Comparison of the effects of remimazolam and dexmedetomidine on early postoperative cognitive function in elderly patients with gastric cancer*” caught our attention.

In this randomized controlled trial, 104 elderly patients undergoing laparoscopic radical resection of gastric cancer were randomly divided into three groups: remimazolam group (Group R), dexmedetomidine group (Group D), and saline group (Group C). The primary outcome was the incidence of early postoperative cognitive dysfunction (POCD). The secondary outcomes were inflammatory response index and S100β protein, the patients' HR and MAP, anesthesia recovery indexes [extubation time, Postanesthesia care unit (PACU) residence time, doses of propofol, and remifentanil], the visual analog scale (VAS) and adverse reactions in the three patient groups, such as respiratory depression, hypotension, bradycardia, agitation, drowsiness, nausea and vomiting. The authors concluded that remimazolam was similarly beneficial as dexmedetomidine in lowering the incidence of early POCD in aged patients after radical gastric cancer resection, probably due to reduced inflammatory response.

Previous investigations have demonstrated that dexmedetomidine can reduce the risk of POCD occurrence (Guo et al., 2021; Xu et al., 2017; Singh et al., 2022). There were also previous studies finding that remimazolam could reduce the risk of POCD (Tan et al., 2022; Huang et al., 2023; Liu et al., 2022). But there were few comparisons between the effects of remimazolam and dexmedetomidine on POCD, the author of this article seized this research gap, conducted this RCT, which has certain clinical guiding significance for the perioperative period management of patients undergoing laparoscopic surgery for gastric cancer. However, this article raised some concerns for us. We believe the conclusions are weak for the following reasons.

## 2 The conclusion is weak

### 2.1 Sample size

The sample size of this study was calculated based on the pre-text results, the incidence of POCD treated by remimazolam is 12%, the incidence of POCD treated by dexmedetomidine is 11%, and the incidence of POCD caused by blank control propofol is 39%, resulting in 35 cases in each group. The relationship between the sample size calculation and the interpretation of the results was that obtaining the sample size calculation for three group rates can only lead to the conclusion that there were differences among the three group rates. Based on the pre-experiment data of this article, the sample size calculation for the comparison of two group rates was also conducted (PASS2021), the result obtained was: R vs. C: resulting in 37 cases in each group; D vs. C: resulting in 34 cases in each group; R vs. D: resulting in 15,973 cases in each group; The sample size of R vs. D was quite different from that of this article. The reason for this gap was that the incidence rates of POCD in group R and group D were very different from that in group C, but the difference in the incidence rate between the two groups was very small. In the other words, the author used the sample size for the comparison of three group rates to obtain the conclusion of the comparison of two group rates, so this conclusion is weak.

### 2.2 The research methods cannot achieve the research purpose

It was written at the end of the preface: The current investigation will contrast the effects of remimazolam and dexmedetomidine on elderly patients undergoing surgery for stomach cancer who experience early postoperative cognitive impairment. And this research purpose corresponded to the title. Facing this research purpose, the most suitable research design is R vs. D, there is no need to add Group C. Because the basic research already has the research results of R vs. C and D vs. C. The comparison of three group rates were mainly used to compare the effective rates of different treatment methods in clinical research. Adding Group C for comparison in this article will instead cause interference to R vs. D. One of the specific manifestations of the interference was the sample size calculation mentioned above.

### 2.3 Data classification extraction error

In table 10, it was the incidence of POCD 1 day after surgery (T5) and 3 days after surgery (T6). But in the description of the results, Yong Qing Liao et al. wrote: at 3 days postoperative (T6) and 7 days postoperative (T7), there was no statistically significant difference (*p* > 0.05) in the incidence of POCD between groups R and D, even though both were lower than the incidence of POCD in group C, which was statistically significant (*p* < 0.05). In the conclusion and outlook section of the article, it was said that the article lacked the statistical data of the incidence of POCD 7 days after surgery, which was consistent with Table 10, but contrary to the description part of the conclusion. In fact, the author calculated the incidence of POCD 7 days after the operation, which is a deficiency in statistical data analysis.

We respectfully appreciate Yong Qing Liao and others for providing us with an important RCT study, which compared the improvement of remimazolam and dexmedetomidine compared with propofol on postoperative cognitive impairment in elderly patients. The author of this review hopes that these comments can improve this article.
